# Endsystolic versus enddiastolic scar imaging for transmurality assessment

**DOI:** 10.1186/1532-429X-13-S1-P158

**Published:** 2011-02-02

**Authors:** Andreas Schuster, Amedeo Chiribiri, Geraint Morton, Masaki Ishida, Kalpa De Silva, Matthias Paul, Shazia Hussain, Divaka Perera, Eike Nagel

**Affiliations:** 1 King's College London, London, UK

## Objective

To investigate the influence of endsystolic scar imaging over routine enddiastolic scar imaging on transmurality.

## Background

The late gadolinium enhancement (LGE) technique has been an important achievement in cardiovascular magnetic resonance (CMR) and is widely used to precisely localize and determine the amount of necrosis and fibrosis. The percentage of transmurality of LGE is inversely related to the likelihood of functional recovery after revascularisation. LGE imaging is usually performed in enddiastole as recommended by current guidelines of the SCMR. Whether or not endsystolic imaging would significantly influence transmurality in patients with ischemic scarring remains unclear.

## Methods

107 segments with moderate hypokinesia or more severe wall motion abnormalities were studied in 20 patients with established coronary artery disease referred for viability assessment (33% of all segments). We used a SSFP standard 4-chamber view to determine the endsystolic and enddiastolic position in the cardiac cycle. LGE imaging was performed with patient specific trigger delays to obtain enddiastolic (LGE_ed_) and endsystolic (LGE_es_) images. Enddiastolic and endsystolic wall thickness (WT_ed_ and WT_es_), thickness of the remaining viable rim (RIM_ed_ and RIM_es_) and thickness of scar in enddiastole was measured manually.

## Results

Evidence of LGE was 84% in all dysfunctional segments with a mean scar of 3.4±2.5 mm. Total wall thickness and the thickness of the remaining viable myocardium increased from diastole to systole (WT_ed_ 7.9±1.9 versus WT_es_ 8.4±2.2, p<0.001 ; RIM_ed_ 4.5±3.1 versus RIM_es_ 5±3.4, p<0.001). There was a difference between the transmurality of scar measured in enddiastole and endsystole (LGE_ed_ 46±33% versus LGE_es_ 44±33%, p<0.001). This difference was most pronounced in a subgroup of segments (n=50) between 25 and 75% transmurality of LGE (LGE_ed_ 57±18% versus LGE_es_ 53±18%, p<0.001). Reduced transmurality was inversely correlated with increased thickness of the remaining viable rim between diastole and systole (r=-0.73).

## Conclusion

Transmurality of scar changes little with varying acquisition times in the cardiac cycle. However there is a statistically significant difference between transmuralities derived from enddiastolic and endsystolic LGE imaging mainly due to the function of the remaining viable rim. Clinically this might not impact on decision making but clearly shows the importance of standardized imaging protocols especially in research studies.

**Figure 1:**
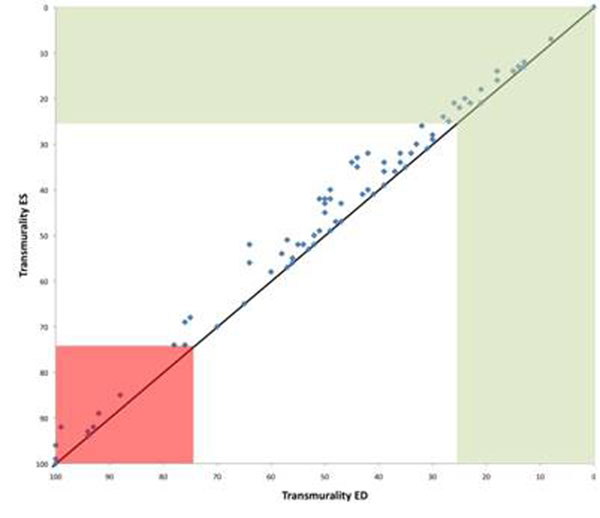
Differences between transmurality in enddiastole and endsystole. Red area indicates transmurality above 75% and green area below 25%.

**Figure 2:**
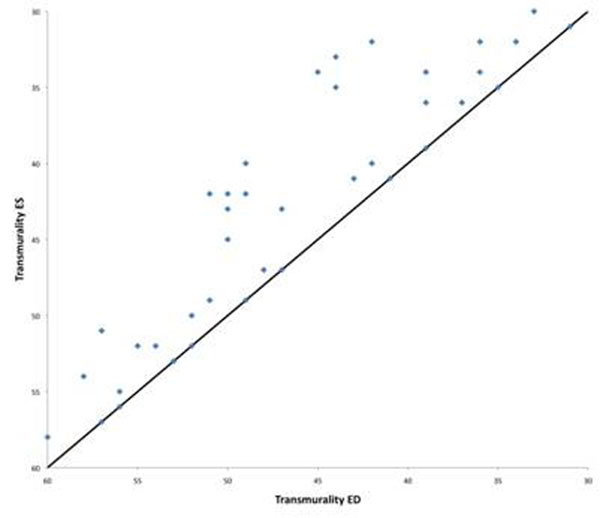
Differences between transmurality in enddiastole and endsystole. Especially in segments with intermediate transmurality this difference can be more extensive.

**Table 1:** 

Enddiastolic versus endsystolic LGE imaging and segmental transmurality	p
All segments (n=107)	

LGE_ed_ 46±33% versus LGE_es_ 44±33%,	<0.001

	

Segments with up to 25% transmurality (n=25)	

LGE_ed_ 25±9% versus LGE_es_ 22±8%	<0.001

	

Segments with 25% and 75% transmurality (n=50)	

LGE_ed_ 57±18% versus LGE_es_ 53±18%	<0.001

	

Segments with transmural scar (n=15)	

LGE_ed_ 99±1% versus LGE_es_ 99±2%	0.55

